# Circ_0004535/miR-1827/CASP8 network involved in type 2 diabetes mellitus with nonalcoholic fatty liver disease

**DOI:** 10.1038/s41598-023-47189-3

**Published:** 2023-11-13

**Authors:** Min Li, Ai Zeng, Xinle Tang, Hui Xu, Wei Xiong, Yanying Guo

**Affiliations:** 1 Graduate School of Xinjiang Medical University, Xinshi District, Ürümqi, 830054 China; 2 B Chao Room, The Sixth Affiliated Hospital of Xinjiang Medical University, Tianshan District, Ürümqi, 830092 China; 3 Department of Laboratory Medicine, The Sixth Affiliated Hospital of Xinjiang Medical University, Tianshan District, Ürümqi, 830092 China; 4 Department of Endocrinology, The Sixth Affiliated Hospital of Xinjiang Medical University, Tianshan District, Ürümqi, 830092 China; 5 Department of Endocrinology and Metabolic Diseases, People’s Hospital of Xinjiang Uygur Autonomous Region, Xinjiang Clinical Research Center for Diabetes Mellitus, Tianshan District, Ürümqi, 830011 China

**Keywords:** Genetics, Biomarkers

## Abstract

Diagnostic delay in type 2 diabetes mellitus (T2DM) with nonalcoholic fatty liver disease (NAFLD) patients often leads to a serious public health problem. Understanding the pathophysiological mechanisms of disease will help develop more effective treatments. High-throughput sequencing was used to determine the expression levels of circRNAs, and mRNAs in health controls, T2DM patients, and T2DM with NAFLD patients. Differentially expressed genes (DEcircRs, DEmRs) in T2DM with NAFLD were identified by differential analysis. The miRNAs with targeted relationship with the DEcircRs and DEmRs were respectively predicted to construct a ceRNA regulatory network. In addition, enrichment analysis of DEmRs in the ceRNA network was performed. The expression of important DEcircRs was further validated by quantitative real-time PCR (qRT-PCR). The steatosis was detected in glucose treated LO2 cells by overexpressing circ_0004535, and CASP8. There were 586 DEmRs, and 10 DEcircRs in both T2DM and T2DM with NAFLD patients. Combined with predicted results and differential analysis, the ceRNA networks were constructed. The DEmRs in the ceRNA networks were mainly enriched in Toll-like receptor signaling pathway, and apoptosis. Importantly, dual luciferase experiments validated the targeted binding of hsa_circ_0004535 and hsa-miR-1827 or hsa-miR-1827 and CASP8. qRT-PCR experiments validated that hsa_circ_0004535, and CASP8 was downregulated and hsa-miR-1827 was upregulated expression in peripheral blood of T2DM with NAFLD patients. Abnormal cell morphology, and increased lipid droplet fusion were observed in the glucose treated LO2 cells, overexpression of circ_0004535 and CASP8 ameliorated these changes. Our work provides a deeper understanding of ceRNA mediated pathogenesis of T2DM with NAFLD and provides a novel strategy for treatment.

## Introduction

Nonalcoholic fatty liver disease (NAFLD) is the most common chronic liver disease and a major health problem, affecting one fourth of the global population^1,2^. NAFLD refers to increased intrahepatic fat accumulation with or without inflammation and fibrosis in the absence of other causes^3^. It can progress to advanced fibrosis, cirrhosis, hepatocellular carcinoma, raising liver related morbidity and mortality^4^. The literature suggests that NAFLD is caused by double hit injury^5^. Additionally, increasing understanding of NAFLD has identified several risk factors associated with this complex hepatic alteration^6^. Among them, insulin resistance (IR) is undoubtedly one of its main features^7^.

Type 2 diabetes mellitus (T2DM) is due to a progressive insulin secretory defect in the context of IR^8^. It poses a continuous and universal threat to human health and healthcare worldwide^9^. Interestingly, recent evidence suggests that T2DM is an independent risk factor for NAFLD^10^. Indeed, NAFLD and T2DM are at the intersection of similar risk factors, epidemiology, and pathophysiology^11,12^. The prevalence of NAFLD in T2DM patients is estimated to be approximately 70%, indicating that NAFLD is closely related to T2DM^13^.

Epidemiological evidence shows a strong bidirectional relationship between T2DM and NAFLD^14^. Clinically, NAFLD coexists with T2DM, which contributes to the severity of NAFLD^15^. In addition, NAFLD and T2DM co-exist and exert synergistic effects leading to poor metabolic status, more severe liver failure and increased risk of cardiovascular disease^16,17^. Therefore, elucidating the pathogenesis of T2DM with NAFLD patients is crucial for managing disease progression and improving patient outcomes.

Over the years, a great deal of work has been done in order to elucidate the pathological mechanisms of T2DM with NAFLD. A recent paper demonstrated that adipose derived microRNAs (miRNAs) regulate gene lists in other tissues such as the liver^18^. A characteristic feature of circular RNAs (circRNAs) is its " miRNA sponge " function, which can effectively bind and inhibit miRNA transcription, further affect downstream mRNA expression, and ultimately participate in various diseases^19^. CircRNAs have been confirmed to be involved in the epigenetic regulation of NAFLD and may be potential therapeutic targets^20^. In addition, circRNA have also been suggested as potential biomarkers for T2DM^21^.

To the best of our knowledge, few studies have focused on the regulatory mechanism of circRNA in T2DM with NAFLD, which is important. In this study, we focused on exploring the potential roles of circRNA mediated ceRNAs in disease by high-throughput sequencing of PBMC samples from patients with both T2DM and NAFLD. Aimed to generate new recommendations for the understanding, diagnosis, and treatment of T2DM with NAFLD patients.

## Materials and methods

### Participants

Peripheral blood samples of participants were collected from the Sixth Affiliated Hospital of Xinjiang Medical University, which included 18 healthy controls, 18 T2DM patients, 18 T2DM with NAFLD patients, and 15 NAFLD patients. Peripheral blood mononuclear cells (PBMC) were isolated from peripheral blood samples using a separation kit (TBD, Tianjin, China). Human studies were reviewed and approved by the Ethics committee of the Sixth Affiliated Hospital of Xinjiang Medical University (No. LFYLLSC20201015-01), and adhered to the Declaration of Helsinki. Written informed consent was obtained from all participants. General data of T2DM patients and T2DM with NAFLD patients is shown in Table 1.

**Table 1:** General data of T2DM patients and T2DM with NAFLD patients (means ± SD).

	T2DM	T2DM with NAFLD	P value
Sex (female/male)	9/9	9/9	> 0.05
Age (years)	55.11 ± 10.70	57.29 ± 9.52	> 0.05
Duration of diabetes (years)	10.31 ± 4.10	8.47 ± 3.86	< 0.05
Duration of NAFLD (years)		3.41 ± 1.68	
BMI (kg/m^2^)	23.55 ± 3.13	28.53 ± 2.87	< 0.05
Current smokers (%)	46.66	53.33	> 0.05
SBP (mmHg)	126.17 ± 16.28	131.94 ± 13.45	< 0.05
DBP (mmHg)	75.24 ± 7.97	81.83 ± 8.27	< 0.05
FBG (mmol/L)	8.12 ± 2.16	8.55 ± 2.10	> 0.05
HbA1c (%)	8.45 ± 2.74	9.31 ± 2.68	< 0.05
TG (mmol/L)	1.61 ± 0.86	2.17 ± 1.03	< 0.05
TC (mmol/L)	4.54 ± 0.79	4.71 ± 1.22	> 0.05

BMI, body mass index; DBP, diastolic blood pressure; FBG, fasting blood glucose; HbA1c, glycated haemoglobin A1c; SBP, systolic blood pressure; TC, total cholesterol; TG, triglycerides.

### RNA extraction and transcriptome sequencing

We randomly selected 9 PBMC samples (3 healthy controls, 3 T2DM patients, and 3 T2DM with NAFLD patients) to extract total RNA using Trizol (Invitrogen, MA, USA). Integrity and concentration of RNA was assessed using the Bioanalyzer 2100 system (Agilent Technologies, CA, USA). A total amount of 3 µg RNA per sample was used and ribosomal RNA was removed with Epicentre Ribo-Zero™ Gold Kit (Epicentre, WI, USA). Then, sequencing libraries were generated according to manufacturers’ instructions with varied index label by NEBNext^®^ Ultra™ Directional RNA Library Prep Kit for Illumina (NEB, Ispawich, USA). Consequently, the libraries were sequenced with the Illumina Hiseq 4000 platform (Illumina) and 150 bp paired-end reads were then generated.

### Data processing

Raw reads were firstly filtered through eliminating adaptor contaminated reads and low-quality reads. Then, clean reads with high quality were chosen to do downstream analyses. For mRNAs, we utilized HISAT2 v2.0.5^22^ to align clean reads to a specified reference genome to obtain positional information on the reference genome or genes and information on sequence features unique to the sequenced samples.

On the other hand, the clean reads were mapped to reference genome and transcriptome using the circBase^23^ to identify circRNAs.

### Quantification of gene expression and differential analysis

The reads numbers were counted using featureCounts v1.5.0-p3. Then FPKM (Fragments Per Kilobase of transcript sequence per Millions) of each mRNA was calculated. The circRNAs expression levels were estimated by number of reads mapping to the backsplice side and quantified by RPM (spliced reads per million reads).

Differential expression analyses for circRNAs and mRNAs between T2DM and control samples, as well as T2DM with NAFLD and controls were performed using the DESeq R package^24^. A *P* value < 0.05 and |log_2_ FC (fold change)|> 2 were assigned as differentially expressed circRNAs (DEcircRs) and differentially expressed mRNAs (DEmRs).

### Construction of the ceRNA network

The targeted regulatory miRNAs of circRNAs were predicted by circbank (http://www.circbank.cn/), miRanda (http://www.microrna.org/), and circinteractome (https://circinteractome.nia.nih.gov/) online websites, respectively. The target mRNAs of miRNAs were predicted using TargetScan (https://www.targetscan.org/vert_80/). Subsequently, target mRNAs were comparatively analyzed with DEmRs. The circRNAs mediated ceRNA networks (DEcircRs/target miRNAs/target DEmRs) were finally constructed and visualized using Cytoscape software (V 3.6.0).

### Enrichment analysis

Enrichment analyses of Gene Ontology (GO) analysis and Kyoto Encyclopedia of Genes and Genomes (KEGG) pathways were performed using clusterProfiler R package^25^ for mRNAs in ceRNA networks. A *P* value < 0.05 was considered as a threshold to determine significant enrichment terms.

### Cell culture and transfection

Normal human hepatocyte cell line LO2 cells were cultured in RPMI1640 medium supplemented with 10% foetal bovine serum, 100 U/mL penicillin, and 100 μg/mL streptomycin in 5% CO_2_ at 37 °C. LO2 cells were also cultured in 10 mM glucose (G1) and 25 mM glucose (G2) RPMI1640 medium supplemented with 10% foetal bovine serum, 100 U/mL penicillin, and 100 μg/mL streptomycin in 5% CO_2_ at 37 °C for 24 h.

The pcDNA3.1-CASP8, pcDNA_NC, pcDNA-circ_0004535, and pcDNA-circ_NC (Genepharma, Shanghai, China) were transformed into LO2 cells separately using Lipofectamine 2000 for 48 h. LO2 cells were then cultured in G2 RPMI1640 medium supplemented with 10% foetal bovine serum, 100 U/mL penicillin, and 100 μg/mL streptomycin in 5% CO_2_ at 37 °C for 24 h.

### Quantitative real-time PCR

Total RNA was extracted from remaining PBMC samples in 15 NAFLD, 15 T2DM, 15 T2DM with NAFLD, and 15 controls, as well as LO2 cells using Trizol kit (Ambion, 15596026). Then RNA was reverse transcribed into cDNA using 5X All-In-One RT MasterMix (ABM, G492). The quantitative real-time PCR (qRT-PCR) was performed using EvaGreen Express 2× qPCR MasterMix-Low Rox (ABM, MasterMix-EL). The 2^−ΔΔCT^ method was used to analyze relative expression. CircRNA and mRNA was normalized to GAPDH, and miRNA was normalized to U6. Primer sequences are shown in Supplemental Table 1.

### Dual-luciferase reporter assay

The binding sites of circRNA-miRNA and miRNA-mRNA were obtained from miRanda and TargetScan. The 3′untranslated region (3′UTR) and the corresponding mutant sequences of CASP8 were cloned into the PmirGLO luciferase reporter vector, termed as CASP8-WT or CASP8-MUT. The 3′UTR and the corresponding mutant sequences of circular RNA hsa-circ-0004535 were cloned into the psiCHECK 2 luciferase reporter vector, termed as circRNA-WT or circRNA-MUT. The 293 T cells were co-transfected with hsa-miR-1827 mimics or hsa-miR-1827 NC (50 nM) and these vectors (50 ng) using the Lipofectamine^®^ 2000 (Invitrogen).

### HE staining and oil red O staining

Cells were fixed with 4% paraformaldehyde for 30 min and washed with PBS for three times. Subsequently, the cells were stained with hematoxylin and eosin following standard protocols. Cells were then sealed with neutral gum and visualized under a microscope (DFC700T, Leica, Germany).

For oil red O staining, cells were stained with oil red O (Solarbio, Beijing, China) for 20 min. Subsequently, Mayer hematoxylin staining solution was added to counterstain the nucleus for 1 min. Covered cells with distilled water and visualized under a microscope (DFC700T).

### ELISA

The triglyceride (TG), activities of superoxide dismutase (SOD), nonesterified fatty acids (NEFA), and malondialdehyde (MDA) were respectively measured using enzyme-linked immunosorbent assay (ELISA) kit (NanjingJiancheng, Nanjing, China) according to the instruction.

### Statistical analyses

Statistical analysis using Prism 5 (GraphPad Software). All data are shown as mean ± standard deviation (SD). Comparisons between groups was analyzed by Student’s *t*-test. *P* < 0.05 was considered statistically significant difference.

### Ethics approval and consent to participate

The study was approved by the ethics committee of the Sixth Affiliated Hospital of Xinjiang Medical University.

## Results

### Differentially expressed genes in T2DM with NAFLD

**Figure 1:**
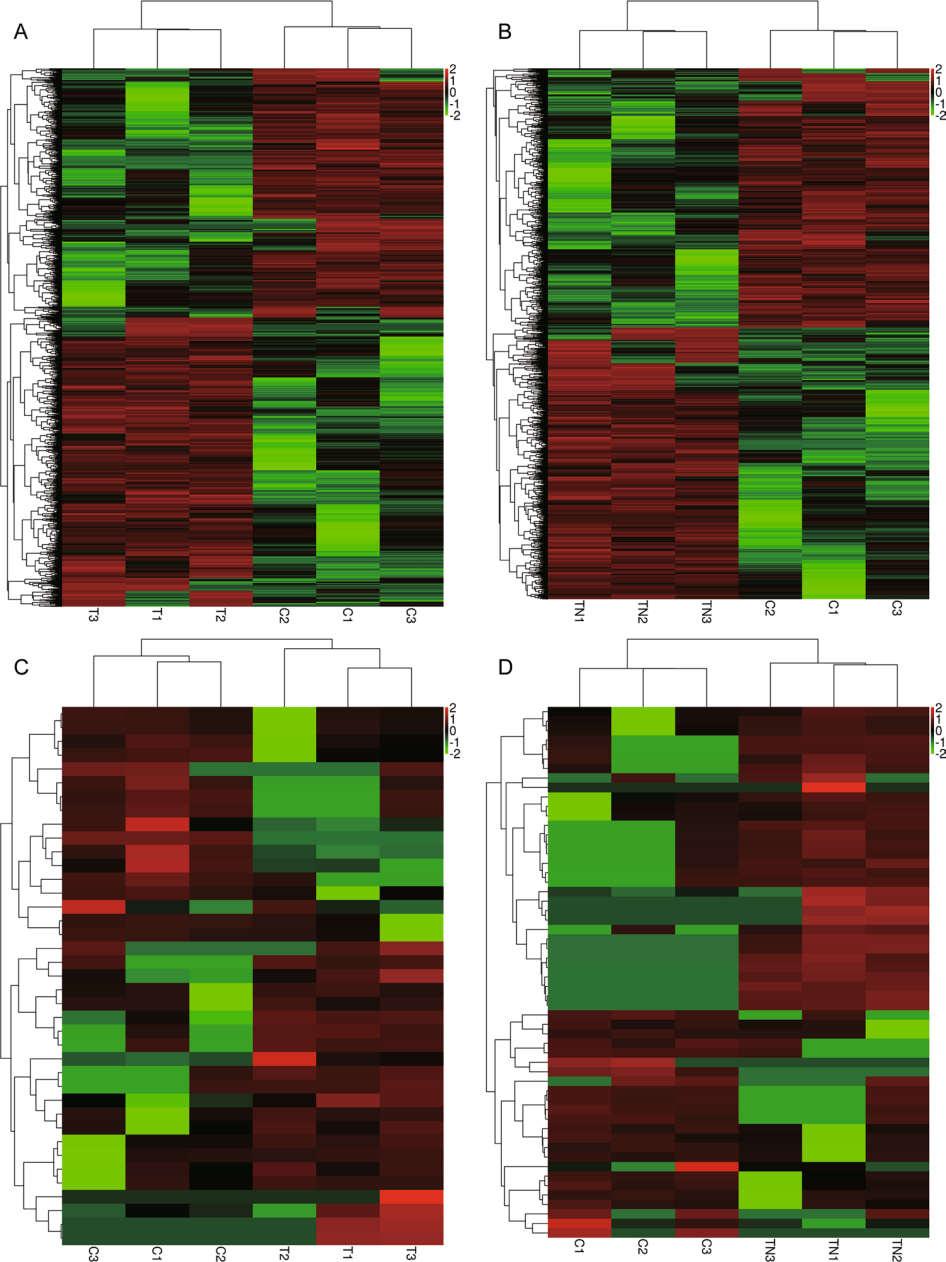
Differentially expressed mRNAs and circRNAs related to T2DM with NAFLD patients. (**A**) Heatmap of differentially expressed mRNAs between T2DM and controls. (**B**) Heatmap of differentially expressed mRNAs between T2DM with NAFLD patients and controls. (**C**) Heatmap of differentially expressed circRNAs between T2DM and controls. (**D**) Heatmap of differentially expressed circRNAs between T2DM with NAFLD and controls. Red is up-regulated expression, and green is down-regulated. C: controls; T: T2DM; TN: T2DM with NAFLD.

To identify abnormalities of gene expression in T2DM with NAFLD, we performed next-generation sequencing on PBMC samples of healthy controls, T2DM, and T2DM with NAFLD. Compared to controls, there were 1411 DEmRs in T2DM (Fig. 1A), there were 1671 DEmRs in T2DM with NAFLD (Fig. 1B). For circRNAs, we found 62 DEcircRs in T2DM (Fig. 1C), and 75 DEcircRs in T2DM with NAFLD (Fig. 1D) compared with controls.

**Figure 2:**
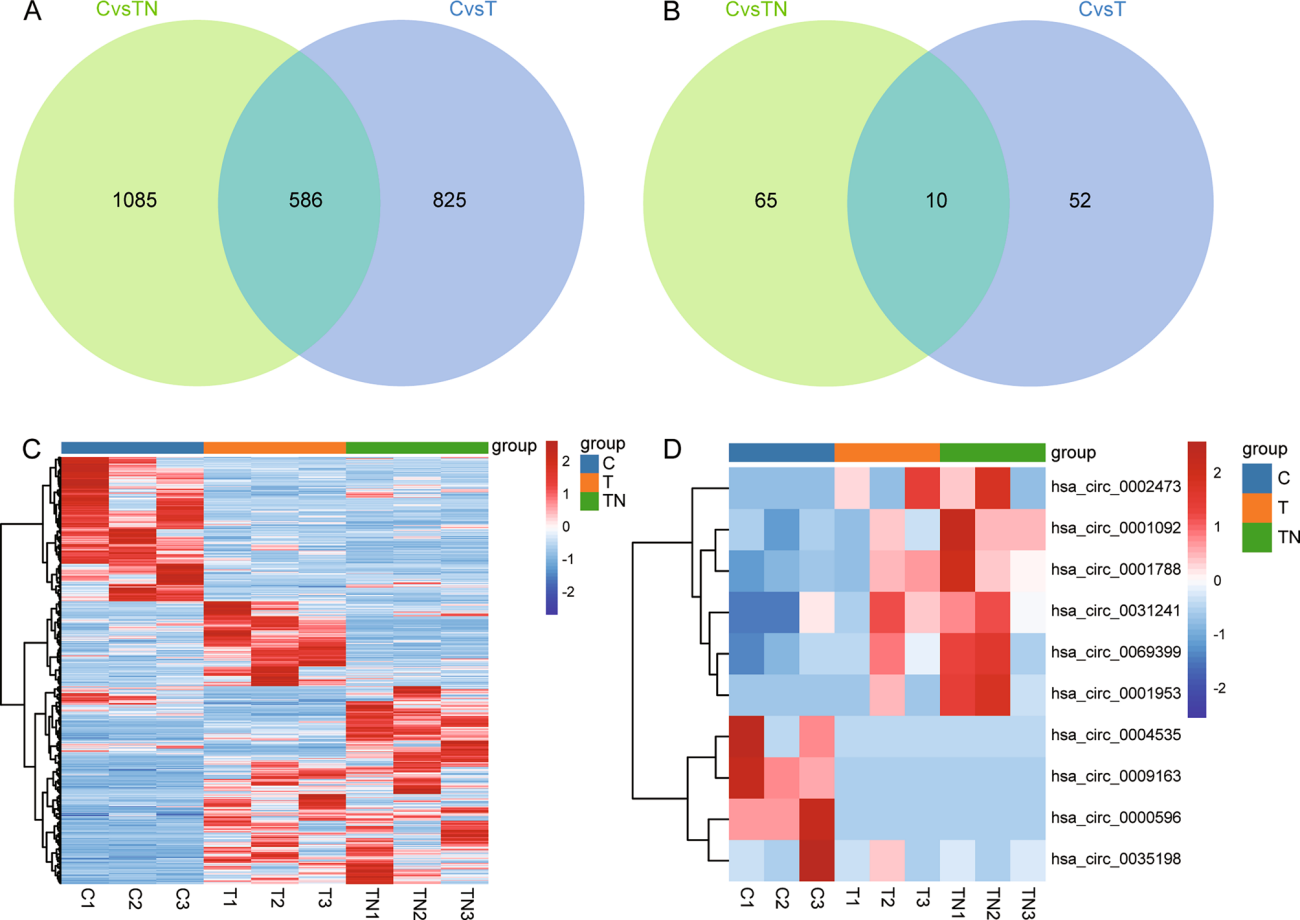
Identify common differentially expressed mRNAs and circRNAs. (**A**) Intersection analysis of differentially expressed mRNAs in T2DM, and T2DM with NAFLD. (**B**) Intersection analysis of differentially expressed circRNAs in T2DM, and T2DM with NAFLD. (**C**) Expression heatmap of common differentially expressed mRNAs in control, T2DM, and T2DM with NAFLD. (**D**) Expression heatmap of common differentially expressed circRNAs in control, T2DM, and T2DM with NAFLD. Red is up-regulated expression, and green is down-regulated. C: controls; T: T2DM; TN: T2DM with NAFLD.

To further characterize genes expression associated with NAFLD and T2DM, we performed an intersection analysis for the two groups of DEmRs or DEcircRs. Finally, we obtained 586 common DEmRs (Fig. 2A) and 10 common DEcircRs (Fig. 2B). The expression of common DEmRs in control, T2DM, and T2DM with NAFLD were shown in Fig. 2C, and the expression of common DEcircRs were shown in Fig. 2D. These genes may be relevant to T2DM with NAFLD.

### Construction of ceRNA networks

**Figure 3:**
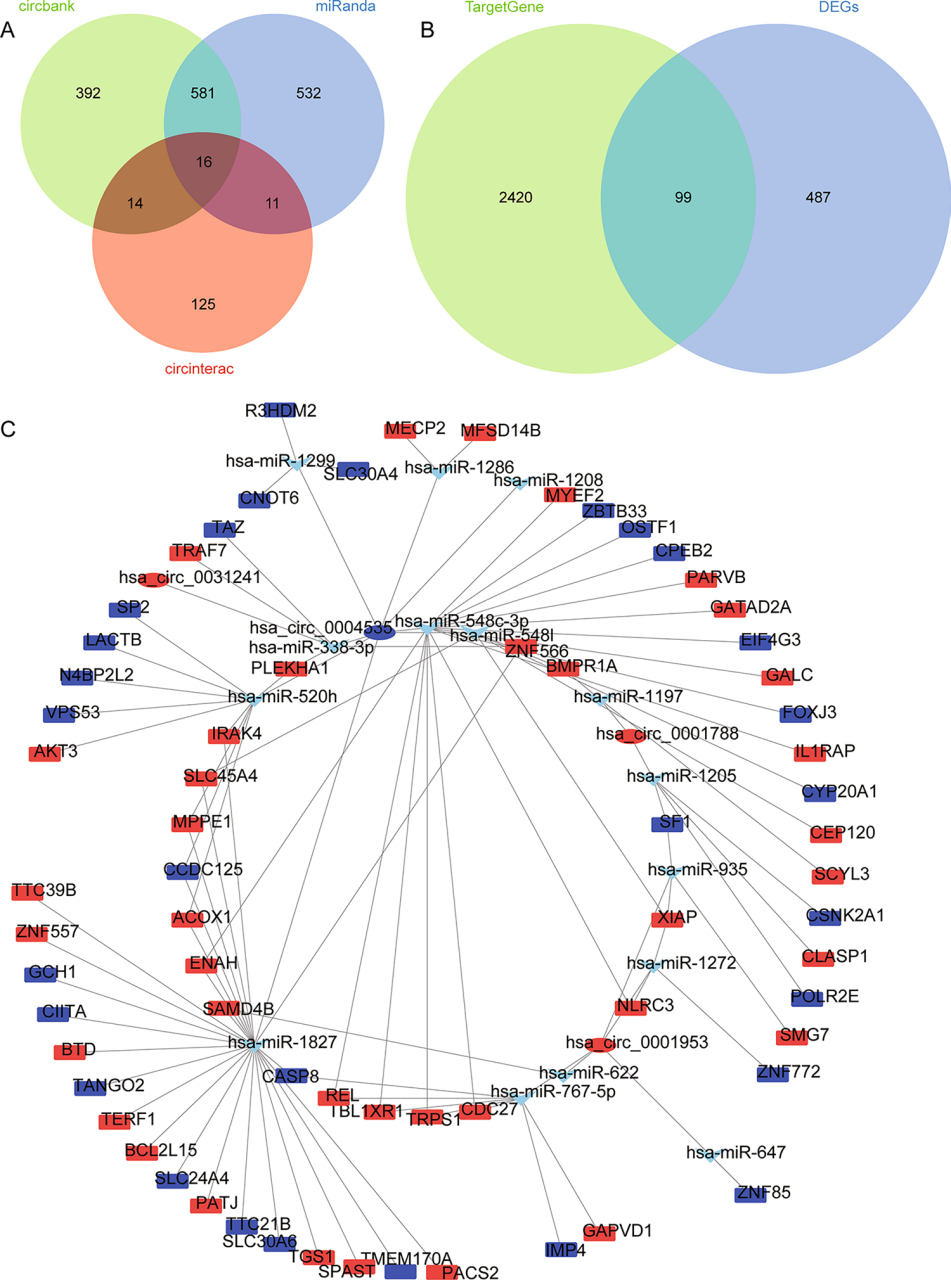
Identification of the ceRNA networks in T2DM with NAFLD. (**A**) The targeted miRNAs of common DEcircRs were predicted using circbank, miRanda, and circinterac websites. (**B**) Intersection between common DEmRs and targeted mRNAs of miRNAs predicted by TargetGene website. (**C**) The DEmRs and DEcircRs with consistent expression direction were screened, and ceRNA network with targeted regulatory relationship was constructed. The network consisting of 4 circRNAs, 15 target miRNAs and their target mRNAs was delineated using Cytoscape software. Red is upregulated gene and blue is downregulated in T2DM, and T2DM with NAFLD.

Through the circbank, miRanda, and circinterac online prediction websites, respectively, we identified a total of 16 miRNAs targeted regulation by the common DEcircRs (Fig. 3A). A total of 2519 target mRNAs of 16 miRNAs were predicted using TargetGene, and 99 target regulated common DEmRs were obtained by comparison with common DEmRs (Fig. 3B). By screening the DEcircRs and target DEmRs with same expression direction, we constructed circRNA-miRNA-mRNA networks (Fig. 3C). The ceRNA networks may have a regulatory role in T2DM with NAFLD.

### Enrichment analysis of DEmRs

**Figure 4:**
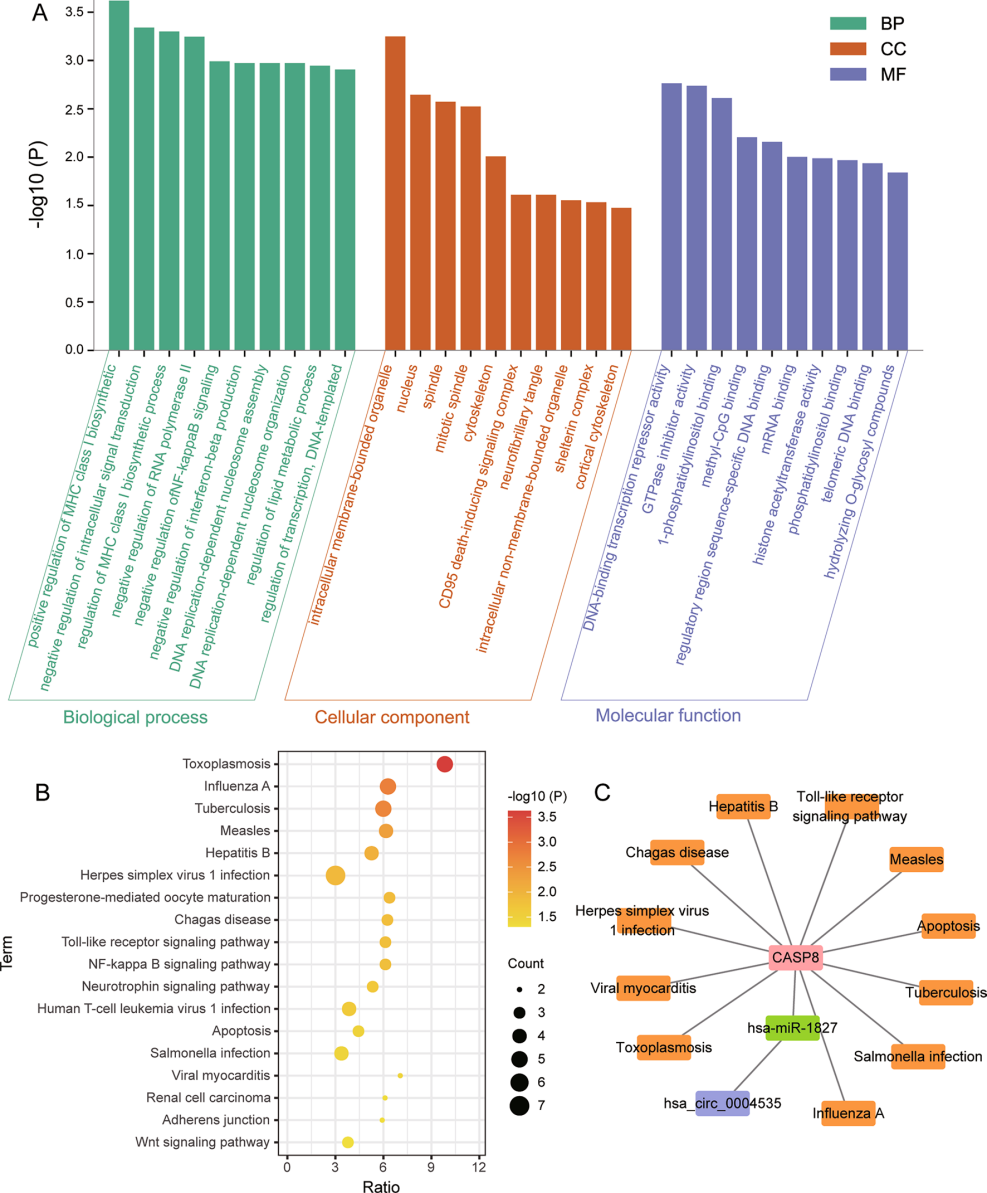
Biological functions of DEmRs in ceRNA networks. (**A**) Classification of top 10 significant GO terms for DEmRs. The results of enrichment cover three sets: biological process, cellular component, and molecular function. (**B**) Significant KEGG pathways for DEmRs. (**C**) The important ceRNA regulatory networks in T2DM with NAFLD.

To explore the underlying mechanism of T2DM with NAFLD, we performed biological functional enrichment analysis of DEmRs in ceRNA networks. The results of GO analysis (Fig. 4A) showed that positive regulation of MHC class I biosynthetic process, negative regulation of intracellular signal transduction, and regulation of MHC class I biosynthetic process were enriched in biological processes (BP). Then, intracellular membrane-bounded organelle, nucleus, and spindle were significantly enriched in cellular component (CC). For molecular function (MF), DNA-binding transcription repressor activity, GTPase inhibitor activity, and 1-phosphatidylinositol binding. In addition, KEGG pathway analysis terms were also significantly enriched, which mainly involved Toll-like receptor (TLR) signaling pathway, and apoptosis (Fig. 4B). Among the DEmRs which involved in KEGG pathways, CASP8 was selected as the core gene due to its involvement in the largest number of pathways. It may play a broader regulatory role in T2DM with NAFLD. Finally, we identified an important ceRNA regulatory network of hsa_circ_0004535/hsa-miR-1827/CASP8 (Fig. 4C). CASP8 was significantly enriched in TLR signaling pathway.

### Experimental validation in patients

**Figure 5:**
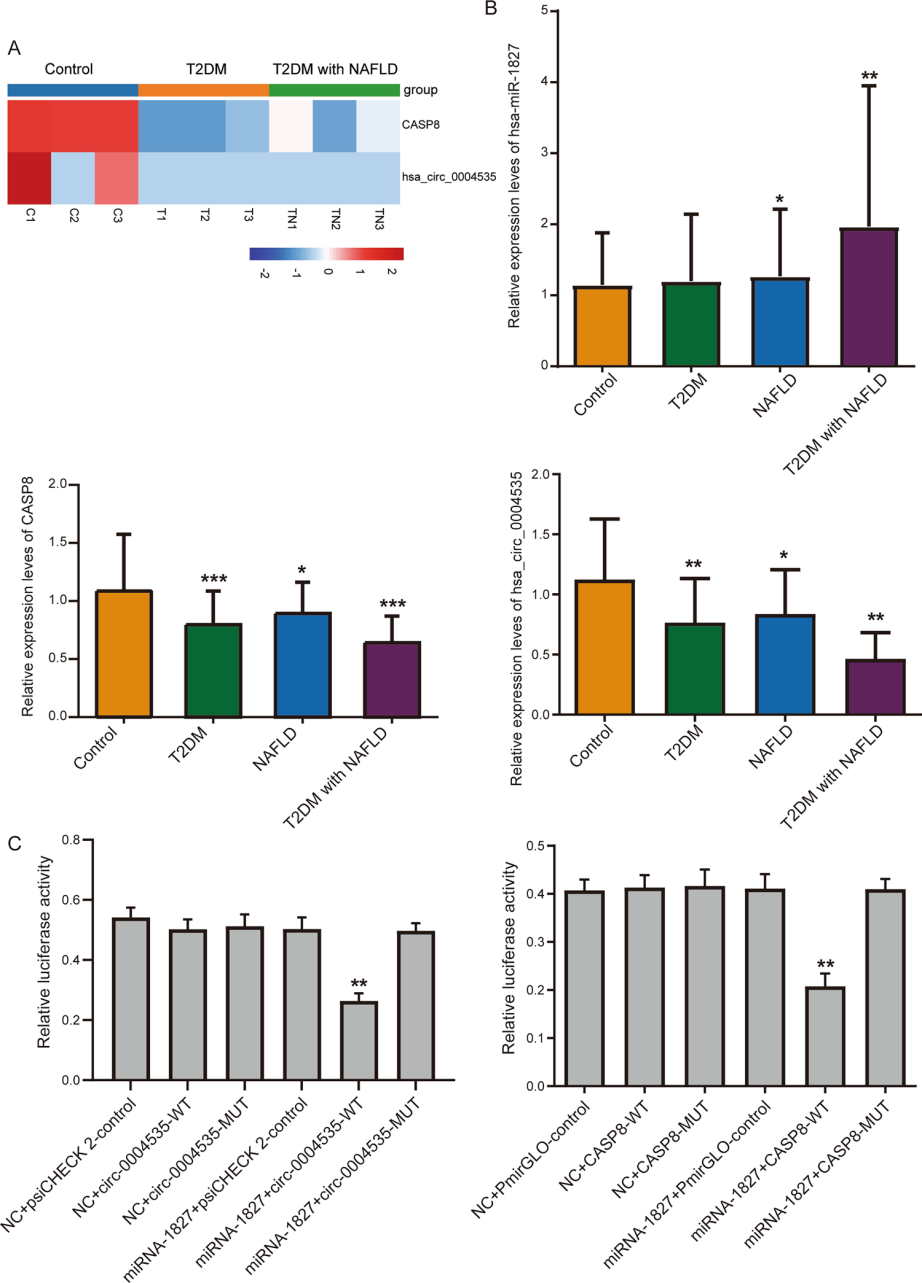
Molecular experiments validate significant analytical results. (**A**) The expression of hsa_circ_0004535 and CASP8 in T2DM, T2DM with NAFLD, and controls. (**B**) Expression changes of hsa-miR-1827, CASP8, and hsa_circ_0004535 in NAFLD, T2DM, T2DM with NAFLD, and controls through qRT-PCR detection. *P < 0.05, **P < 0.01, ***P < 0.001. (**C**) Relative luciferase activity in 293 T cells co-transfected with the hsa-miR-1827 mimics or NC and circRNA-WT or circRNA-MUT (left); the luciferase activity in 293 T cells co-transfected with the hsa-miR-1827 mimics or NC and CASP8-WT or CASP8-MUT (right). **P < 0.01.

Through the differential analysis results, we found that the expression of hsa_circ_0004535 and CASP8 in the T2DM or T2DM with NAFLD was all lower than that in the controls (Fig. 5A). To further investigate the significant results we obtained, we performed molecular experiments in clinical samples. We utilized qRT-PCR experiments to examine expression levels of hsa_circ_0004535, hsa-miR-1827, and CASP8 in NAFLD, T2DM, T2DM with NAFLD, and controls (Fig. 5B). The results showed that hsa_circ_0004535, and CASP8 were expressed at lower levels in NAFLD and T2DM patients than in controls, and even lower in T2DM with NAFLD patients, while hsa-miR-1827 was higher expression in T2DM with NAFLD than others.

Afterward, to identify ceRNA network with possible regulatory roles in T2DM with NAFLD, we performed dual luciferase experiments (Fig. 5C). As expected, the luciferase activity of 293 T cells co-transfected with hsa-miR-1827 mimics and circRNA-WT was significantly weaker than that co-transfected with hsa-miR-1827 mimics and circRNA-MUT. Weaker luciferase activity was also detected in the 293 T cells co-transfected with hsa-miR-1827 mimics and CASP8-WT. This prompted that hsa-miR-1827 and circular RNA hsa-circ-0004535, as well as hsa-miR-1827 and CASP8 had complementary binding sequences.

### Circ_0004535/miR-1827/CASP8 affects steatosis of LO2 cells

**Figure 6:**
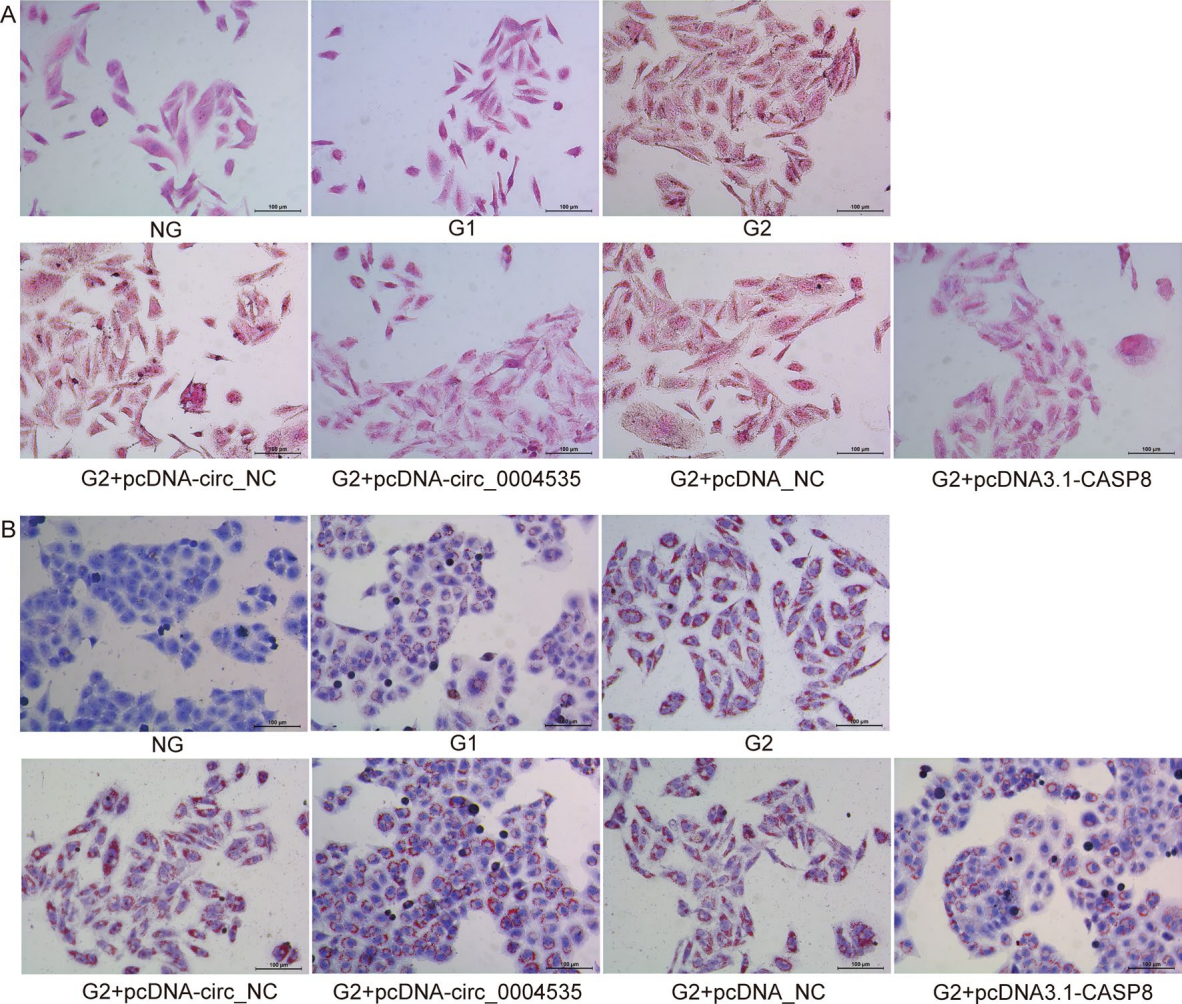
Effect of overexpression circ_0004535 and CASP8 on pathological changes in high glucose treated LO2 cells. (**A**) Representative HE-stained LO2 cells. (**B**) Representative Oil Red O-stained LO2 cells. Scale bar = 100 µm.

HE staining and Oil Red O staining (Fig. 6A,B) showed that compared to the NG group, cells in the G2 group had diffused edges, unclear internal structures, and lipid droplet fusion. Compared with the NC group, overexpression of circ_0004535 and CASP8 notably improved the cell structure, and the red lipid droplets were significantly reduced.

**Figure 7:**
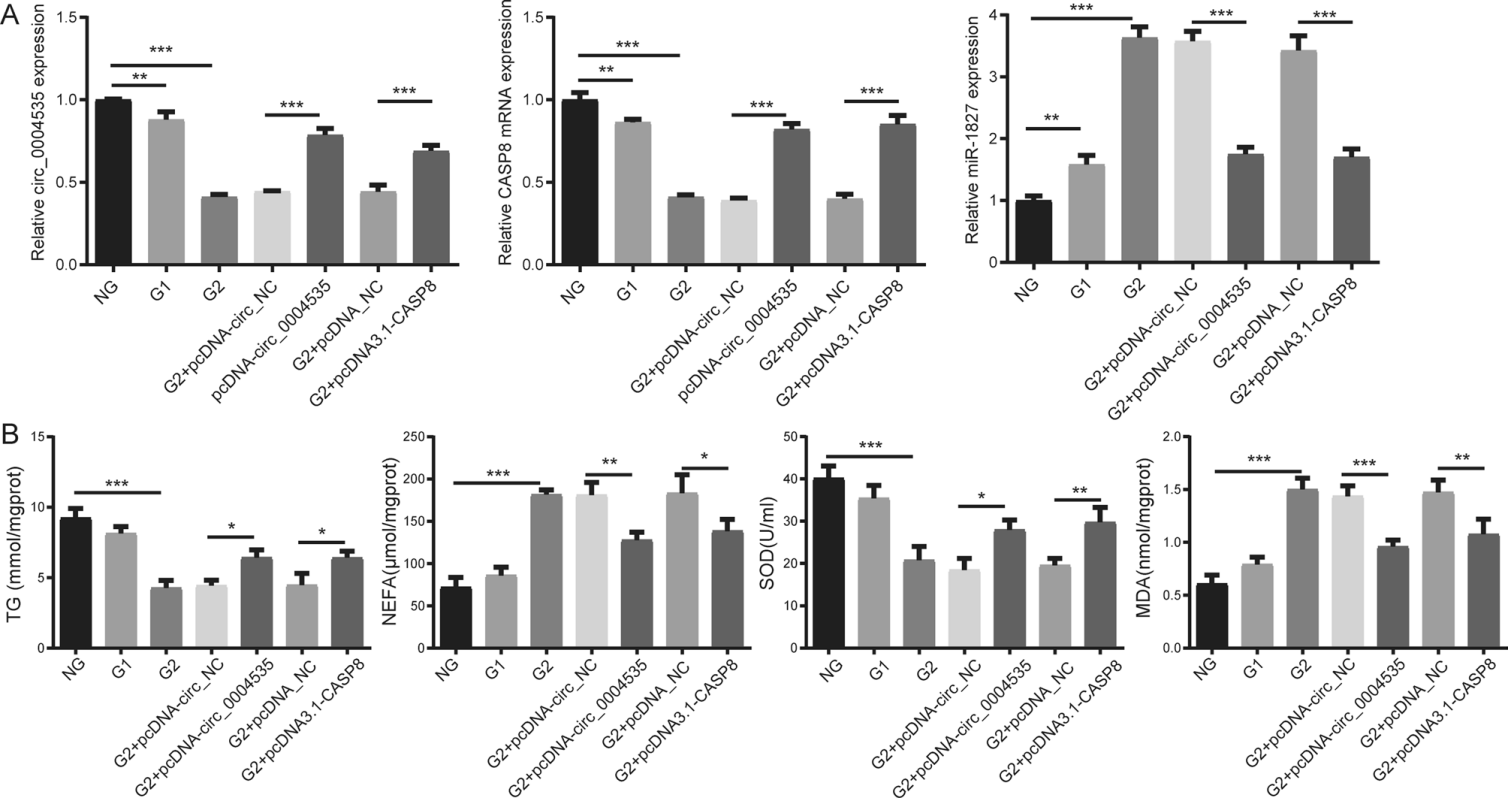
Overexpression circ_0004535 and CASP8 improves high glucose treated LO2 cells. (**A**) The mRNA levels of ceRNA network, including circ_0004535, hsa-miR-1827, and CASP8. (**B**) The content of NEFA, MDA, TG, and SOD. *P < 0.05, **P < 0.01, ***P < 0.001. TG, triglyceride; NEFA, nonesterified fatty acids; SOD, activities of superoxide dismutase; MDA, malondialdehyde.

Moreover, compared with the NG group, the expression of circ_0004535 and CASP8 in G2 group was significantly reduced, while the expression of hsa-miR-1827 significantly increased; Compared with the NC group, overexpression of circ_0004535 and CASP8 increased the expression of circ_0004535 and CASP8, and decreased the expression of hsa-miR-1827 (Fig. 7A). In the G2 group, the content of NEFA, and MDA significantly increased, while TG, and SOD content significantly decreased. Compared with NC group, overexpression of circ_0004535 and CASP8 increased the content of TG, and SOD, and reduced the content of NEFA, and MDA (Fig. 7B).

## Discussion

T2DM and NAFLD share the same pathophysiology and nearly identical risk factors and share the same prevention and management strategies^26^. In the present study, we explored the aberrant expression of circRNAs and mRNAs in T2DM with NAFLD patients. The ceRNA network of circRNA-miRNA-mRNA and potential regulatory mechanisms were constructed. Notably, circ-0004535 participated in the pathological process of T2DM with NAFLD by interacting with miR-1827, downregulating CASP8 expression, regulating TLR signaling pathway and apoptosis.

An increasing number of studies have shown that circRNAs are closely associated with the pathogenesis of T2DM and NAFLD^27,28^. However, the understanding of the exact roles and molecular mechanisms of circRNAs in T2DM with NAFLD patients is still in its infancy. Our study identified differential circRNAs and mRNAs in healthy controls, T2DM patients and T2DM with NAFLD. We identified 75 circRNAs that were simultaneously differentially expressed in T2DM patients and T2DM with NAFLD patients. KEGG enrichment analysis results showed that the mRNAs of the ceRNA network were mainly involved in the TLR signaling pathway, and apoptosis. TLRs are key players in the inflammatory response and serve to drive inflammatory cytokine production and may also contribute to pancreatic islets β Cell dysfunction, subsequently leading to T2DM and NAFLD^29,30^. MyD88 is one of the most important adaptor proteins in TLR signaling, and when MyD88 knockout mice are fed a high-fat chow diet, insulin and cholesterol levels increase and liver dysfunction appears, which are all associated with T2DM and NAFLD^31^. Elevation of inflammation and oxidative stress promotes pancreatic islet β Apoptosis of cells, enhancing insulin resistance and thus accelerating the progression of T2DM and NAFLD^32,33^.

Numerous studies have shown that circRNAs can act as miRNA sponges and in turn regulate mRNA expression^34^. In the present study, we identified a ceRNA network of hsa_circ_0004535/hsa-miR-1827/CASP8 may have an important regulatory role in T2DM with NAFLD. Studies in a variety of tumors have found that miR-1827 affects patient prognosis by regulating the expression of downstream target genes through binding to their 3'UTRs^35–37^. MiR-1827 by targeting PPAR- δ involved in the T2DM disease process^38^. In addition, the upstream apoptosis initiator caspases 8 (CASP8) are activated upon receiving a death signal from the death inducing signaling complex (DISC), which in turn activates the downstream apoptotic effector caspases 3, 6 and 7, which ultimately execute apoptosis^39^. CASP8 is a key suppressor of nonalcoholic steatohepatitis (NASH) metabolic disorders and a therapeutic target for T2DM patients^40,41^. CASP8 is also associated with inflammatory pathways and immune cell infiltration in T2DM^42^. Although no studies have been reported so far, the results of our analysis are suggestive of hsa_circ_0004535 and hsa-miR-1827 are associated with T2DM with NAFLD.

Previous studies have shown that circular RNAs in the cytoplasm are more stable than corresponding linear isoforms^43^, making circRNAs attractive biomarkers for human diseases. Consistent with the sequencing analysis, qRT-PCR analysis confirmed that hsa_circ_0004535 was downregulated expression in T2DM, NAFLD, T2DM with NAFLD patients. We also conducted a set of experiments to understand the role and impact of circ_0004535 and CASP8 in the regulation of cell structure and lipid metabolism. The findings suggest a critical role of circ_0004535 and CASP8 in maintaining cellular structural integrity and lipid homeostasis^44,45^. The increase of NEFA and MDA, and the decrease of TG and SOD levels in the G2 group, possibly indicating lipid metabolism disruption and oxidative stress^46,47^. However, circ_0004535 and CASP8 overexpression reversed these trends, further indicating their role in maintaining lipid homeostasis and redox balance in the cells.

The present study has some limitations. The abnormal expression of ceRNA network needs further validation in a larger cohort. Moreover, it is necessary to probe the correlations between ceRNA network of hsa_circ_0004535/hsa-miR-1827/CASP8 and clinical features of T2DM with NAFLD patients.

## Conclusion

In conclusion, the present study shows that hsa_circ_0004535 is expressed at lower levels in T2DM, NAFLD, T2DM with NAFLD patients compared with controls. Furthermore, hsa_circ_0004535 targeted binding hsa-miR-1827 regulated CASP8 expression and participated in TLR and apoptosis related functions. The ceRNA network of hsa_circ_0004535/hsa-miR-1827/CASP8 in T2DM with NAFLD patients and the underlying mechanism needs further investigation. This ultimately contributes to early prediction of disease as well as therapeutic intervention.

### Supplementary Information

Supplementary Table S1.

## Data Availability

The data used in this study are available in PRJNA885850 of BioProject.
