# Supplementary material for: Circ_0004535/miR-1827/CASP8 network involved in type 2 diabetes mellitus with nonalcoholic fatty liver disease
Source: Sci Rep. 2023 Nov 13;13:19807. doi: 10.1038/s41598-023-47189-3 (PMC10643362; doi:10.1038/s41598-023-47189-3)
Supplement: Supplementary file 1 — Supplementary Table S1. [file 41598_2023_47189_MOESM1_ESM.docx]

Table S1. Sequences of primers.

| Gene | Sequence (5′->3′) | Accession number |
| --- | --- | --- |
| hsa_circ_0004535-F | ACCAGCCCCAAAGATAACTGA | NR_034089.1 |
| hsa_circ_0004535-R | ATGAACCACCACGTCTAGCC |  |
| CASP8-F | GTTGGAGGAAAGCAATCTGT |  |
| CASP8-R | TTGAGCCCTGCCTGGTGTCT |  |
| hsa-miR-1827-F | ACACTCCAGCTGGGTGAGGCAGTAGA | NR_031728.1 |
| hsa-miR-1827-R | CTCAACTGGTGTCGTGGAGTCGGCAATTCAGTTGAGATTCAATC |  |
| U6-F | CTCGCTTCGGCAGCACA |  |
| U6-R | AACGCTTCACGAATTTGCGT |  |
| GAPDH-F | CTCGCTTCGGCAGCACA |  |
| GAPDH-R | AACGCTTCACGAATTTGCGT |  |
